# Supplementary material for: Tune out pain: Agency and active engagement predict decreases in pain intensity after music listening
Source: PLoS One. 2022 Aug 3;17(8):e0271329. doi: 10.1371/journal.pone.0271329 (PMC9348657; doi:10.1371/journal.pone.0271329)
Supplement: S1 File — (PDF) [file pone.0271329.s001.pdf]

The Analgesic Potential of Music for Pain

Study Information

Hypotheses

Hypothesis 1: Increased perceived control predicts decreases in pain intensity and pain unpleasantness.

Hypothesis 2: There will be a difference in pain scores between people in the low music complexity and high music complexity conditions.

Hypothesis 3: There will be a difference in aesthetic responses in terms of emotion, enjoyment, and engagement between the low music complexity and high music complexity conditions.

Design Plan

Study type

Experiment - A researcher randomly assigns treatments to study subjects, this includes field or lab experiments. This is also known as an intervention experiment and includes randomized controlled trials.

Blinding

For studies that involve human subjects, they will not know the treatment group to which they have been assigned.

Is there any additional blinding in this study?

This study is being run online so no personnel will come into contact with participants.

Study design

We have a 2 x 2 between groups experimental design with two levels for choice (no choice and perceived choice) and two levels for music (high complexity and low complexity).

No files selected

Randomization

Participants will be randomized via block randomization to one of the four music listening conditions via the Qualtrics platform.

Sampling Plan

Existing Data

Registration prior to creation of data

Explanation of existing data

No response

Data collection procedures

Participants will be recruited via the Prolific participant online recruitment survey platform. A short screening questionnaire will be used to identify eligible participants, according to the inclusion and exclusion criteria below. If participants indicate that they are eligible they will be invited to log in later on the same day to complete the full survey. Participants will be asked to confirm that they are still experiencing pain for the second survey. Participants will be offered payment at a rate of £9 per hour, increasing to a rate of £10 per hour if there is a slow recruitment rate.

Inclusion Criteria:
- Over 18 years of age
- Access to audio and headphones to listen to music
- Currently feeling pain
- Report pain intensity to be of at least 2 on a pain intensity Visual Analogue Scale that ranges from 0 'no pain' to 10 'worst pain imaginable'.

Exclusion Criteria:
- Currently on prescription medication for pain management
- Has taken prescription or non-prescription pain medication in the last 8 hours
- Has been experiencing pain for 6 months or longer

No files selected

Sample size

Our target sample size is 329 participants. We will attempt to recruit 362 under the assumption that not all will complete the total task.

Sample size rationale

For Hypothesis 1 and Hypothesis 2:
An a priori sample size calculation using G*Power indicates a sample size of 329 participants will be required for a 2 x 2 ANCOVA analysis with 6 covariates to achieve 0.95 power based on an effect size of f = 0.23 in line with the analgesic effect of music identified in a previous meta-analysis (Lee, 2016). Attrition will be monitored as we recruit participants and we may collect additional participants to make sure we reach the minimum value (e.g. if 10% attrition, then an additional 33 participants would be recruited).

For Hypothesis 3:
An additional sample size calculation was conducted in relation to the analysis for hypothesis 3. The calculation was conducted using r software, using the pwr package with samplesize_mixed function and design_effect function extensions from strengejacket/sjstats on github. The calculation indicates a sample size of 288 participants will be required to achieve power of 0.85 for a linear effects model with an effect size of f= 0.23, with 4 degrees of freedom for the numerator and an interclass correlation of 0.05. The sample size indicated above will be sufficient to conduct this analysis.

Stopping rule

N/A

Variables

Manipulated variables

Music: Music will have two levels.
High Complexity. The main track is a bespoke piece of music created for this study, specifically composed to reduce pain at a population level. The specific music brief is included on OSF wiki.
Low Complexity. An alternative version of the main track has been produced by the same artist using the same musical structure and melody as the main track but with less instrumentation and sound effects, leading to a comparable track but with lower complexity .

Choice: Perceived level of music choice will have two levels.
No choice. Participants will listen to one 3s clip before listening to the same song in full.
Perceived choice from four songs. Participants will be naïve to the fact that they are listening to four 3s clips from different parts of the same song. Participants will be asked to choose between the four options but the final "chosen" song is predetermined by the experimenter in each condition.

No files selected

Measured variables

Dependant Variables
Pain intensity: "How much pain do you feel now?"; 100-point visual analogue scale with two anchor points "no pain at all" (0), and "worst possible pain" (100). Measured before and after music listening.

Pain unpleasantness: "How unpleasant is the pain now?"; 100-point visual analogue scale with two anchor points ranging from "not unpleasant at all" (0), to "extremely unpleasant" (100). Measured before and after music listening.

Analgesic potential: "How much do you think this music is helpful to reduce pain?" 100-point visual analogue scale with two anchor points "it does not help at all" (0) to "It helps a lot" (100). Measured after music listening.

Responses to music
Emotions evoked by the music: 9-item Geneva Emotional Musical Scale (GEMS-9; Zentner et al., 2008) rated on a five point Likert scale (1 = "not at all"; 5 = "very much").

Attention and Enjoyment subscale from the Cognitive Vitality Questionnaire, an instrument specifically designed to record responses to music in pain management contexts. This questionnaire has been designed and used by the two main authors in a previous study and demonstrates a good factor structure and reliability. Responses are collected on 100-point visual analogue scales.

Individual variables - These measures will be included as co-variates*
*Before items are included they will be checked for high levels of correlation.

Well-being. Participants will complete the 4-item HowRu Health Status (Benson et al., 2010) and 4-item personal well-being measure (Benson et al., 2019). Items are rated on a 4-point Likert scale.

Trait Empathy. The 7-item fantasy subscale of the Interpersonal Reactivity Index (IRI; Davis, 1980) will be used as an indicator of trait empathy. The fantasy subscale taps respondents' tendencies to transpose themselves imaginatively into the feelings and actions of fictitious characters in books, movies, and plays. Participants will rate each item on a five-point Likert scale (1 = "does not describe me well"; 5 = "describes me very well").

Emotional Engagement with Music. The emotional engagement subscale from the Goldsmiths musical sophistication index v1.0 (GMSI; Müllensiefen et al., 2014) will be used to measure emotional engagement with music. Participants will respond on a 7-point Likert scale (1 = "completely disagree"; 7 = "completely agree").

Musical anhedonia. The Barcelona Music Reward Questionnaire (Mas-Herrero et al., 2013) will be used to assess musical anhedonia. Participants will respond on a 5-point Likert scale (1 = "completely disagree"; 5 = "completely agree").

Perceived self-efficacy. Participants will rate their agreement with the following item on a 10-point scale ranging from 1 (strongly disagree) to 10 (strongly agree): I am confident in my ability to solve problems that I might face in life (For example: I can usually handle whatever comes my way; If I try hard enough, I can overcome difficult problems) (Williams & Smith, 2016).

Locus of control. Participants will respond to Kovaleva's (2012) four-item scale for the assessment of locus of control (IE-4) on a five-point Likert scale (1 = "does not apply to me at all"; 5 = "applies completely").

Pliance. Participants will respond to two-items with high factor loadings from the generalized pliance questionnaire (Ruiz et al., 2019) on a seven-point Likert scale (1 = "never true"; 7 = "always true").

No files selected

Indices

Scores for each standardized measure will be calculated in accordance with the relevant scoring procedures (items will be reverse scored as required).

Individual items for emotional responses to music will be added to provide an index of 'emotional intensity'.
Mean scores for the Attention and Emotion subscale will be computed to provide an index of 'emotional intensity'.

No files selected

Analysis Plan

Statistical models

To address Hypothesis 1 and Hypothesis 2:
A 2x2 factorial ANCOVA will be used to compare the four different treatment arms using SPSS. The manipulated categorical variables are 'Music' and 'Choice'. The dependent variable is pain, measured in terms of pain intensity and pain unpleasantness. A separate ANCOVA analysis will be completed for each of the dependent variables. Individual characteristics and individual responses to the music will be included in the analysis as covariates. Residuals of the linear model will be examined to identify if the assumptions for the linear model were met (i.e., linearity, normality, and homoscedascity).

To address Hypothesis 3:
We will use linear mixed effects models to analyse participant responses to the two music tracks. The manipulated categorical variables are 'Music' and 'Choice' and these will be included in the model as fixed factors. Individual participant responses will be included as a random factor. The dependent variable is Enjoyment and Attention. Residuals of the linear model will be examined to identify if the assumptions for the linear model were met (i.e., linearity, normality, and homoscedascity). Individual characteristics will be included in the analysis as covariates.

No files selected

Transformations

If the residuals do not meet the assumptions for linear model (i.e., linearity, normality, and homoscedascity) a log transformation will be conducted on the data, and the data will be re-analysed.

Inference criteria

Hypothesis 1 and 2
We will use the standard $p < .05$ criteria, combined with 95% confidence intervals, and standard errors for determining if the ANCOVA and the post-hoc test suggest that the results are significantly different from those expected if the null hypothesis were correct. The post-hoc Tukey-Kramer test adjusts for multiple comparisons.

Hypothesis 3
We will use the $p < .05$ criteria, combined with 95% confidence intervals, standard errors and intraclass correlations for determining if the linear mixed model and subsequent pairwise comparisons suggest that the results are significantly different from those expected if the null hypothesis were correct.
Multiple comparisons will be accounted for in the linear model using the least significant difference.
The Akaike Information Criterion will be used to select the most appropriate linear model model. Residuals will be inspected and plotted to determine the quality of the model.

Data exclusion

Data from participants who fail attention checks in the Qualtrics survey will be excluded. Participants with a completion time +/-3 SD of the mean completion time will be excluded.
Otherwise, outliers will be included in the analysis.

Missing data

Participants who fail to complete the two main pain measures (pain intensity and pain unpleasantness) and the music listening task will not be included in the analysis.

Exploratory analysis

The composer created the main track to imbue a sense of wonder in participants and the alt track was identified to reduce the likelihood that participants would experience a sense of wonder.

Other

Other

References

Basiński, K., Zdun-Ryżewska, A., Greenberg, D. M., & Majkowicz, M. (2021). Preferred musical attribute dimensions underlie individual differences in music-induced analgesia. Scientific Reports, 11(1), 1-8. https://doi.org/10.1038/s41598-021-87943-z
Benson, T., Sizmur, S., Whatling, J., Arikan, S., McDonald, D., & Ingram, D. (2010). Evaluation of a new short generic measure of health status: HowRu. Journal of Innovation in Health Informatics, 18(2), 89-101.http://dx.doi.org/10.14236/jhi.v18i2.758
Benson, T., Sladen, J., Liles, A., & Potts, H. W. (2019). Personal Wellbeing Score (PWS)—a short version of ONS4: Development and validation in social prescribing. BMJ open quality, 8(2), e000394. http://dx.doi.org/10.1136/bmjoq-2018-000394
Bradt, J., Potvin, N., Kesslick, A., Shim, M., Radl, D., Schriver, E., Gracely, E. J., & Komarnicky-Kocher, L. T. (2015). The impact of music therapy versus music medicine on psychological outcomes and pain in cancer patients: a mixed methods study. Supportive Care in Cancer, 23(5), 1261-1271. https://doi.org/10.1007/s00520-014-2478-7
Davis, M. H. (1983). Measuring individual differences in empathy: Evidence for a multidimensional approach. Journal of Personality and Social Psychology, 44(1), 113-126. https://doi.org/10.1037/0022-3514.44.1.113
Howlin, C., & Rooney, B. (2021). Cognitive agency in music interventions: Increased perceived control of music predicts increased pain tolerance. European Journal of Pain. https://doi.org/10.1002/ejp.1780
Kovaleva, A. (2012). The IE-4: Construction and validation of a short scale for the assessment of locus of control. (GESIS-Schriftenreihe, 9). Köln: GESIS - Leibniz-Institut für Sozialwissenschaften. https://doi.org/10.21241/ssoar.37119
Lee, J. H. (2016). The effects of music on pain: A meta-analysis. Journal of Music Therapy, 53(4), 430-477. https://doi.org/10.1093/jmt/thw012
Mas-Herrero, E., Marco-Pallares, J., Lorenzo-Seva, U., Zatorre, R. J., & Rodriguez-Fornells, A. (2012). Individual differences in music reward experiences. Music Perception: An Interdisciplinary Journal, 31(2), 118-138. https://doi.org/10.1525/mp.2013.31.2.118
Müllensiefen, D., Gingras, B., Musil, J., & Stewart, L. (2014). The musicality of non-musicians: an index for assessing musical sophistication in the general population. PloS ONE, 9(2), e89642. https://doi.org/10.1371/journal.pone.0089642
Ruiz, F. J., Suárez-Falcón, J. C., Barbero-Rubio, A., & Flórez, C. L. (2019). Development and initial validation of the Generalized Pliance Questionnaire. Journal of Contextual Behavioral Science, 12, 189-198.https://doi.org/10.1016/j.jcbs.2018.03.003
Tsai, H. F., Chen, Y. R., Chung, M. H., Liao, Y. M., Chi, M. J., Chang, C. C., & Chou, K. R. (2014). Effectiveness of music intervention in ameliorating cancer patients' anxiety, depression, pain, and fatigue: A meta-analysis. Cancer Nursing, 37(6), E35-E50. https://doi.org/10.1097/ncc.0000000000000116
Williams, G., & Smith, A. P. (2016). Using single-item measures to examine the relationships between work, personality, and well-being in the workplace. Psychology, 7, 753-767. http://dx.doi.org/10.4236/psych.2016.76078
Zentner, M., Grandjean, D., & Scherer, K. R. (2008). Emotions evoked by the sound of music: characterization, classification, and measurement. Emotion, 8(4), 494-521. https://doi.org/10.1037/1528-3542.8.4.494

Links to sample size calculation package:
https://github.com/strengejacke/sjstats/blob/master/R/samplesize_mixed.R
https://strengejacke.github.io/sjstats/

Contributors
Claire Howlin, Brendan Rooney, and Alison Stapleton

Description
The importance of personal choice in music listening interventions for pain management has been replicated across several high-quality studies and meta-analyses (Bradt et al., 2015; Basiński, et al., 2021; Lee, 2016; Tsai, et al., 2014). More recently, the role of perceived choice has been shown to increase pain tolerance in an experimental setting using the cold pressor task as a proxy for pain (Howlin & Rooney, 2021). The main aim of this study is to test the extent to which the finding that perceived control of music increases pain tolerance extends beyond a lab setting. This study will also explore whether there are additional analgesic benefits from music that is specifically designed and composed to relieve pain at a population level. In line with models of empirical aesthetics, music complexity will be examined as a mediator of analgesic benefits, due to enhanced engagement mediated by optimal complexity. Finally, this study will examine the role of individual attributes, namely musicality, empathy, and age, in mediating the analgesic benefits for pain.
Show less

Registration type
OSF Preregistration

Date registered
July 26, 2021

Date created
July 26, 2021

Associated project
osf.io/4ywjd

Internet Archive link
https://archive.org/details/osf-registrations-egqaz-v1

Category
Project

Registration DOI
10.17605/OSF.IO/EGQAZ

Publication DOI
No publication DOI

Subjects
Psychology
Social and Behavioral Sciences
Cognition and Perception
Health Psychology
Pain Management

Affiliated institutions
This registration has no affiliated institutions

License
CC-By Attribution 4.0 International

Tags
Agency
Choice Experiments
Music
Music Complexity
Pain

Citation
osf.io/egqaz

Copyright © 2011-2022 Center for Open Science | Terms of Use | Privacy Policy | Status | API
TOP Guidelines | Reproducibility Project: Psychology | Reproducibility Project: Cancer Biology
