# Supplementary material for: Tune out pain: Agency and active engagement predict decreases in pain intensity after music listening
Source: PLoS One. 2022 Aug 3;17(8):e0271329. doi: 10.1371/journal.pone.0271329 (PMC9348657; doi:10.1371/journal.pone.0271329)
Supplement: Attachment — Submitted filename: Response to Reviewers.docx [file pone.0271329.s002.docx]

Dear Dr. Natar,

We sincerely thank the editor and the reviewer’s for their careful consideration of this manuscript, and would like to extend our appreciation for the time and effort taken to do this. The suggestions and comments from the review team have been most helpful to improve the clarity of reporting.

After careful consideration, we have decided to revise the paper in light of the recommendations made by the two reviewers. We have attached point by point responses to the issues raised as requested, and we hope you will find these to be satisfactory.

Additionally we have amended the title to reflect the final results more closely.

Yours Sincerely,

Claire Howlin

1. The research gaps should be further specified, based on empirical research, and the novelty and the benefit of the study should be more emphasized in the introduction.

**Response:** On further consideration, the authors agree with the reviewers that a more nuanced and extended consideration of the literature needs to be included, and each of the points below have been addressed.

a. Regarding the role of control, the authors mention only one study on pain tolerance – which they actually don’t measure themselves. Other studies did not show that participants’ own choice of music (full control) would be associated with stronger analgesic effects than music chosen by others. A more differentiated view would be necessary.

**Response:** A number of experimental and clinical studies demonstrate the importance of personal choice, and personal agency in mediating the analgesic benefits of music listening have been included in the introduction. The revised document also clarifies that the aim is to extend the previous findings beyond pain tolerance to explore the pain experience. Line 65 - 69 and 78-79

b. The role of music features is widely under-researched, thus general statements about their weak impact should be avoided. It should be clarified if complexity has already been investigated in the context of analgesic effects of music listening or not. Interpretation (s. abstract, conclusions) should not be generalized on music features as a whole, but only on the investigated feature (complexity, evtl. mixed with tempo, s. 12).

**Response:** Several studies have tested if basic acoustic properties or if combinations of musical features of music are directly related to the analgesic benefits of music engagement, without much success. The previous version of the document overlooked these and the revised document now elaborates on the previous literature exploring the role of musical features in mediating the analgesic benefits for pain has been included from Line 37 - 46.

c. The authors highlight active engagement and assume that higher levels of musical sophistication may be associated with stronger pain-reducing effects. However, they don’t explain enough why they assume so and if they assume this only for active engagement or also the other sub-components. This and the research gaps should be further clarified.

**Response:** In the revised document, we now put forward an argument based on the cognitive vitality model that outlines why higher cognitive processes such as cognitive agency and active cognitive engagement with music should be considered to understand how music can help to reduce pain. Line 48 - 59. Additionally a more explicit link is made between individual characteristics and music engagement. line 96-106.

2. The hypotheses should target the research gaps and should be specified, mainly hypotheses 2 and 3. In hypothesis 2, the role of complexity should become clear. In hypothesis 3, it should become clear which individual attributes are meant. The introduction suggests that musical sophistication or active engagement are targeted, but more individual attributes are investigated. It is not clear if an interaction between complexity and engagement is expected.

**Response:** The hypotheses have been revised in line with the comment above. Indeed, our pre-registered hypotheses were paraphrased in the introduction (but not the discussion) in the previous document. The revised document includes the elaborated pre-registered hypotheses. Line 114 - 124

3. In line with 2., the title lets assume that several individual attributes and features are targeted, but the introduction doesn’t focus on several ones. This should be adapted.

**Response:** In the revised manuscript the individual traits are introduced more explicitly in the introduction. The title has also been changed. Line 96-106

4. In line with 2., in the methods section, more parameters are introduced than the theoretical introduction lets assume (e.g. emotional responses and other individual traits than active engagement). Also, it is not always clear how and when variables were assessed. It should become clear which parameters are investigated as primary outcome variables for the investigation of the main hypotheses, and which parameters are additionally investigated and for what reasons, including how and when they were assessed.

**Response:** All of the individual characteristics are now introduced in the introduction of the revised manuscript. Line 96-106. In addition, the revised “*Research Questions and Hypotheses*” section now clarifies the rationale for their inclusion and their relative importance in the research. Line 130 - 133.

5. Apart from the research ethics exemption, it is not specified whether the authors followed any ethical guidelines. A data monitoring section including where and how long data is stored, who has access and how confidentiality is ensured is missing.

**Response:** An extended ethics statement and declaration of data storage procedures has now been included in the revised text. In addition it can be added that the anonymous data were collected using a secure online platform which is compliant with Data Protection regulations and downloaded to a cloud-based university server, as an encrypted zip file protected by a strong password. Line 147 - 152.

6. With regard to the study design, the researchers should clarify why they decided for an experimental online setting on persons with different types of pain instead of a controlled laboratory experiment and/or a pre-defined type of pain.

**Response:** We previously conducted a lab-based experiment using a cold pressor task to test the role of cognitive agency, musical features and individual attributes in mediating the analgesic benefits of music listening. One of the core aims of this study was to test if the findings that we had observed in the lab would hold in a real world context. Line 78 - 78, and 139 - 142.

7. It is unclear why the authors examined a sample size of 286 subjects. Was the group size based on expected effect sizes? Was a power analysis performed? Was it a convenience sample?
**Response:** Here we pre-registered our recruitment strategy and the inclusion/exclusion criteria before collecting data. We then followed these steps. The revised document now includes further details in relation to the a priori power calculation, and decision for stopping based on the observed power. Only participants who met the full inclusion criteria were included. Line 153 - 161.

8. A control group with no music or an alternative stimulus presentation is missing. Decreases in pain intensity and unpleasantness can therefore not be clearly attributed to the music, but only comparisons between the groups can be interpreted. The general effects (and effect sizes) of music listening should be interpreted with more caution.

**Response:** The lack of a control group with no music has been highlighted as a limitation of the study, and references are made in the text at the level of the group rather than directly to the music. Line 45-459

9. The sample is very heterogenous. Different types of pain, gender, countries, languages, general medication intake, physical diseases, psychological disorders, body mass index, drug consumption, pregnancy, and music-related profession do not seem to be checked or controlled for. Since these parameters can potentially influence pain perception, the interpretation should be done with much more caution.

**Response:** While not all of these variable were controlled for, the use of random assignment to the conditions means that it is extremely unlikely that the groups differ systematically on any of these variables. For these reasons it is typical in experimental design to treat the groups as comparable in these regards. Inferential statistics are used in the analysis to estimate the impact of these “noise” variables and remove them from the estimated models as “error” variance. What remains are the statistically significant effects of the systematically measured variables. It is these variables (choice, complexity and active engagement) upon which we make inferences and claims.

Nonetheless, several factors were taken into consideration when screening participants for inclusion, including drug consumption, and pregnancy. The revised manuscript includes a more extensive description of the recruitment procedure and inclusion criteria on lines 120 -134. One of the core aims of this study was to replicate a finding that was previously demonstrated in lab setting, to a real world clinical sample. For this reason we felt it was appropriate to include a relatively heterogenous sample to represent different types of acute pain, to increase the generalisability of the findings.

10. It should be specified how participants were recruited, why a pain rating of 2 in the NRS, and pain for less than 12 weeks were inclusion criteria, if for any of the points mentioned in 9. was controlled for, and, if measured, how the groups can be described with regard to these points.

**Response:** These inclusion/exclusion criteria were specified in the pre-registration before data were collected. The text has been amended to highlight that a pain rating of 2 was used in the inclusion criteria 'to ensure that they were experiencing at least a mild level of pain before music listening', line 171 and that 'pain extending beyond 12 weeks is often classified as chronic pain (31)' according to the international classification of diseases (ICD-11) line 173..

11. The instructions regarding the process of music listening should be specified.

**Response:** The instruction given to participants in the process of music listening is now included in the revised manuscript. Line 191 - 193.

12. It is not clear why the composed music conditions differ besides complexity also in tempo. For the investigation of complexity, an additional difference in tempo is not necessary and might limit the interpretability of the results. The reasons for this approach should be explained.
**Response:** The variation in tempo is unavoidable, given that extra percussion was added to the track to increase the complexity, the perceived tempo also increases - even though the core structure is unchanged. To highlight the great care that was taken to minimise the difference between the two versions, while achieving a perceivable difference. The revised manuscript has been amended to reflect this at line 256-259.

13. The authors write in a table note that one item of the GMSI Active Engagement scale was deleted because it did not load well. Given that the authors mention that the GMSI is validated, this approach is unusual. Changing the scale may affect validity. A citation should be given to support this approach, and validity should in best case be checked before interpreting the results from the new scale. The change should be described in the main text. Instead of citing a book for the validation of the GMSI, the research papers should be cited directly. Regarding fig. 2, it should become clear how the cut-off values for Active Engagement were chosen.

**Response**: Checking the validity of a scale would not be possible based on the data collected. However, construct validity can be inferred from the reliability of the measure. It was indeed this analysis that identified the poor loading of one item. It was removed to as to increase the construct validity of the measure. The plot lines fitted to the data are used to illustrate the degree to which the relationship between choice and pain changes depending on different levels of active engagement. The description for figure 2 has been updated to indicate this more clearly.

14. More details regarding the multilevel modelling are necessary: Were pain intensity and unpleasantness included in one model or separate models?

Was the model fit evaluated with a specific parameter?

Were all possible effects included and then non-significant effects deleted, or were effects included step-by-step?

Were all possible interaction effects investigated or only specific ones, what were the reasons? It is not clear why age was included as a predictor – its role should be introduced based on research.

**Response:** The revised manuscript now includes more details about the multilevel modelling approach taken in the data analysis section line 284 - 294..

15. The results of the different multilevel models (at least null model and final model) should be made available for the reader, and more parameters are necessary. For an example and recommendations on multilevel modelling, see e.g.: Aguinis, H., Gottfredson, R. K., & Culpepper, S. A. (2013). Best-practice recommendations for estimating cross-level interaction effects using multilevel modelling. Journal of Management, 39, 1490-1528.

**Response:** The revised manuscript now reports the results of the multilevel models according to the best practice guidelines recommended by the reviewer, and now includes information about the null model and additional parameters in table 3 and table 4.

16. General recommendations for the therapeutic context should not be given. Too little is known about interpersonal differences regarding perception, traits, and (emotional) reactions.

**Response:** General recommendations for therapeutic contexts have been replaced with the relevance of these findings to future clinical and experimental studies has been highlighted. Line 458 - 459.

17. Limitations are missing in the discussion section.

**Response:** The discussion has been amended to reflect the limitations of the study more clearly from line 447 to 454.

18. It should become clear earlier that an online study was conducted.

**Response:** The first section of the methods section now indicates that this study was conducted online. "The experiment was presented online using the Qualtrics platform on line 184.

19. A description of potential dropped out participants and how was dealt with them in the analyses is missing.

**Response:** From an initial sample of 585 participants that completed the survey, 286 people completed all components of the experimental procedure. The revised manuscript includes a more extensive description of the recruitment procedure and inclusion criteria on lines 120 -134.

20. Block randomization should be described in more detail regarding its settings/criteria.

**Response:** The term Block randomisation was misused in error and the details of simple randomisation to independent groups is now clarified.

‘using the Qualtrics randomiser, participants were randomly allocated to one of the four conditions:’

21. Regarding the manipulation check of complexity, another term than “Aesthetic responses” should be chosen because complexity was rated and not the aesthetic evaluation. It should be specified how complexity was evaluated by the participants.

**Response:** A number of ratings were collected to determine the aesthetic appeal of the music including complexity, enjoyment, boredom, interest, attention in line with Berlyne's model of aesthetic engagement. The revised manuscript has now been amended to reflect this from line 311 - 315.

22. In the discussion, the authors write that repeated-measures experiments would not be possible in an acute pain context. However, repeated measures studies are recommended for pain research due to strong interindividual differences in pain perception. In order to prevent carry-over effects, longer time periods between the measures are necessary.
**Response:** The discussion has been amended to reflect more clearly that the between participants design is a limitation of this study on line 452-459.

23. L. 217/218: “Low in boredom” is not equivalent to “aesthetically pleasing”.

**Response:** A number of ratings were collected to determine the aesthetic appeal of the music including complexity, enjoyment, boredom, interest, attention in line with Berlyne's model of aesthetic engagement. The revised manuscript has been amended to highlight this from line 306 - 310.

24. L. 237 and following lines: Wording of the hypotheses should be consistent.

**Response:** the wording of the hypotheses has now been amended to be more consistent.

Reviewer #2: The manuscript presents an experimental online study of music induced analgesia in individuals suffering from acute pain conditions. Overall I think that this is an interesting and important study, yet more detailed information is needed in the manuscript to assess its methodological and scientific merits.

The study was performed online in a group of 286 adults experiencing acute pain from various causes. I feel that the studied group needs to be described in more detail in terms of demographics (gender information is missing), as well as in terms of the pain-related metrics. Were there any participants that were recruited but did not finish the study? Did they differ in terms of individual characteristics from participants that finished the study? Additionally, I think that with this kind of online methodologies, there is a significant possibility of introducing all kinds of biases. The manuscript would benefit from a section of the discussion outlining these. Another problem is the huge heterogeneity of pain conditions (back pain, headache, joint pain, neck pain, period pain etc.). This heterogeneity may be in part the cause of large individual variance that has been reported and was not controlled for in the statistical model. I feel that this should also be addressed in the discussion.

**Response:** The revised manuscript now includes additional details (including gender and pain type) about the overall sample and the composition of each group has been provided in the demographics section and table 1. Additionally more detailed information has been included in relation to our screening and recruitment procedure for the study on Line 120-134. The use of random assignment to the conditions means helps to ensure that the groups do not differ systematically on any of these variables. For these reasons it is typical in experimental design to treat the groups as comparable in these regards. Inferential statistics are used in the analysis to estimate the impact of these “noise” variables and remove them from the estimated models as “error” variance. What remains are the statistically significant effects of the systematically measured variables. It is these variables (choice, complexity and active engagement) upon which we make inferences and claims. One of the core aims of this study was to replicate a finding that was previously demonstrated in lab setting, to a real world clinical sample. For this reason we felt it was appropriate to include a relatively heterogenous sample to represent different types of acute pain, to increase the generalisability of the findings.

Furthermore, I am not sure about the phrasing of the hypotheses 2 and 3:

- "Hypothesis 2: There are analgesic benefits from music specifically designed and composed to
maximise individual engagement." In comparison to? Music not maximising individual engagement? I'm not sure if the study addresses this hypothesis. Perhaps it is a matter of phrasing the hypothesis differently.

- "Hypothesis 3: individual attributes related to musicality predict analgesic responses to music for acute pain" Musicality? From what I understand, it was not tested here. Or did you mean active engagement or sophistication?

**Response:** The hypotheses have been revised in line with the comment above. Indeed, our pre-registered hypotheses were paraphrased in the introduction (but not the discussion) in the previous document. The revised document includes the elaborated pre-registered hypotheses that clarify the issues raised. Line 114 - 124

Also, the Statistical analysis and Results sections need more clarification:

- What was the rationale behind using a mixed model instead of a regular linear model? Did the authors compare ratings before and after music, independently for each of the experimental conditions? Or did they compare "pain reduction scores" (pre-music pain - post-music pain)? This should be explained more thoroughly.

**Response:** The revised manuscript now includes more details about the multilevel modelling approach taken, and the rationale for this approach in the data analysis section line 279 - 289. Further clarification has also been included to explain how the pain reduction scores were calculated on line 279 - 280.

- Descriptive statistics for pain intensity and unpleasantness are much needed. I would also advise including a plot that visualizes the distributions of pain scores and their changes in response to music in each of the experimental conditions.

**Response:** The revised manuscript now includes descriptive statistics for pain reduction scores in terms of pain intensity and pain unpleasantness on line 296 - 300.. Violin plots have also been included that visualise the distributions of pain scores and their changes in response to music in each of the experimental conditions on line 285-286.

- A discussion of statistical power and sample size justification would be appropriate.

**Response:** Here we pre-registered our recruitment strategy and the inclusion/exclusion criteria before collecting data. We then followed these steps. The revised document now includes further details in relation to the a priori power calculation, and decision for stopping based on the observed power. Only participants who met the full inclusion criteria were included. Line 153 - 161.

- I don't understand why the authors decided to use only the Active Engagement subscale of the GMSI and exclude other GMSI subscales from the models.

**Response:** A top-down linear modelling strategy with a loaded matrix structure was used. Items that did not significantly contribute to the best model fit were removed from the model. The active engagement sub-scale was the only sub-scale of the GMSI that significantly contributed to the best fitting model.

- In Table 2, do I understand correctly that for unpleasantness, no interactions were tested? Why?

**Response:** A top-down linear modelling strategy with a loaded matrix structure was used. Items that did not significantly contribute to the best model fit were removed from the model. Unpleasantness interactions did not significantly contribute to the best fitting model, and so they were removed from the final model.

- I don't think that the conclusion that "The present study also replicated the finding that choice is more important than music features..." is valid. The present study addressed one music feature (complexity) by manipulating it systematically. Other musical features (ie. melody, harmony, timbre, genre, production style, syncopation) were not considered but may influence MIA. This conclusion is also apparent in the title of the paper and I think it gives a wrong impression about what has been tested in this study.
**Response:** The conclusion has been amended to contextualise the current findings within a growing consensus that supports the argument that music features are not as important as personal choice. line 475 - 485. Additionally the title has been amended to be more precise.

Other considerations:

- L180: What about the running time of the low-complexity track? Was that the same as high complexity? If not, the effects could be attributed to different running times.

**Response:** The manuscript has been amended to indicate that " both versions of the track were kept quite short (3 minutes and 24 seconds) on line 260.

- How do the differences in emotional responses to high vs low complexity music influence the results? Emotion is an often discussed factor in MIA. I feel this should be addressed in the discussion.

**Response:** Although participants provided slightly different emotional responses in the two different music conditions, this did not result in differences in pain responses. The text has been amended to note this on line 404 - 410.

- The OSF repository cannot be freely accessed, as if it has not been made public. This might be intentional, although as a reviewer, I would gladly look at the preregistration report.
**Response:** The OSF repository is now publicly available at and we invite the reviewers to look at the preregistration report. https://osf.io/egqaz

- I don't think I understand the point in the discussion about repeated measures (L336-340). Why is it a limitation if the present study was not using a repeated manipulation?

**Response:** The discussion has been amended to reflect more clearly that the between participants design is a limitation of this study on line 452 - 459.

- Line 62: Might want to check the reference to Brattico & Pearce, 2013

**Response:** The reference to Brattico & Pearce, 2013 has been updated into the correct format.

- L88: "At the same time, this study will explore how individual levels of the trait Active Engagement in the general population relate to the analgesic benefits from music listening."
Why general population? Perhaps this sentence should be phrased differently.

**Response:** This statement has been amended *'At the same time, this study will explore how individual levels of the trait Active Engagement relate to the analgesic benefits from music listening.'*

- L200: "linear modelling was used because the intraclass correlation was .34, which indicates the need for multilevel modelling" according to whom? A citation is needed here.

**Response:** Further clarification has been included in the revised manuscript and an additional references were added to support this approach. Line 284 - 294.

- L233: "This indicates that mean pain ratings went from being classed as moderate pain to minor pain." According to which classification? Can we say that for all participants, or is this just a group mean score? Overall, I'm not sure if this is justified.

**Response:** Upon recommendation from the reviewers this section has been removed.

- In the methods section, it would be great to see brief descriptions of the subscales of GMSI.

**Response:** The revised manuscript includes brief descriptions of the subscales of the GMSI on line 229 - 234.

- L263: "Together these findings indicate that the role of cognitive agency and active engagement play a bigger role in reducing pain intensity compared to pain unpleasantness." Please check for grammar.

**Response:** The text has been amended "Together these findings indicate that the role of cognitive agency and active engagement are more likely to reduce pain intensity ratings compared to pain unpleasantness." Line 384-386.

- L365: "The current study furthers these findings by extending them beyond the laboratory to a sample who are experiencing real acute pain and by demonstrating that these effects remain when the music is completely unfamiliar to participants." Please check grammar.

**Response:** The text in the current manuscript now reads 'The current study extends previous findings that demonstrate the importance of cognitive agency beyond a laboratory setting to a sample who are experiencing real acute pain'. Line 485-487.
