# Supplementary material for: Tune out pain: Agency and active engagement predict decreases in pain intensity after music listening
Source: PLoS One. 2022 Aug 3;17(8):e0271329. doi: 10.1371/journal.pone.0271329 (PMC9348657; doi:10.1371/journal.pone.0271329)
Supplement: Attachment — Submitted filename: Response to Reviewers.docx [file pone.0271329.s003.docx]

Response to reviewers.

We thank each of the reviewers for their time and attention on this manuscript. In response to the reviewers requests we have added further clarification sand details on several points, supplied further details on the data analysis and have re-organised where all of these details are found, and carefully reviewed and revised the entire manuscript to eliminate typos and minor errors. We hope that you will agree that the manuscript has been further strengthened it terms of clarity and structure, and should be of high interest to the readers of PLoS one.

Thank you for agreeing and taking the time to complete this additional review. Below you will find a point by point response to each of the queries raised.

1. The authors improved the introduction in different aspects. One issue should still be considered in order to strengthen the introduction even more. The authors focus on findings on pain tolerance, and aim to extend these findings on everyday acute pain experience. In the part of research questions and hypotheses it turns out that pain intensity and unpleasantness are investigated as the main outcome variables of interest but these parameters are not mentioned before. I recommend to introduce pain intensity and unpleasantness as well as potential existing or a lack of existing findings on these important outcome variables already in the introduction of the manuscript.

**Response:** The introduction now introduces the concepts of pain unpleasantness and pain intensity, and the rationale for using these measures to measure pain. Line 120 - 127.

2. The research questions and hypotheses are now clearer. Three issues should still be considered in this section:

2a. The authors write that it would be important to make sure that the music tracks are comparable in terms of “aesthetic and emotional responses” (lines 125-126). However, in the next sentence, the third hypothesis only targets the “aesthetic response”, even though both aesthetic and emotional responses are investigated separately. This should be adapted.

**Response** This section of the text has now been amended (Line 143 - 144).

2b. I highly appreciate the mentioned pre-registration of the project and its hypotheses. However, even though the project is specified as public, the downloadable zip-folder is empty in the given link to the webpage of [osf.io](http://osf.io). This is also important regarding the data availability.

**Response:** There seems to be a technical issue with the OSF repository, on our side we can see the review at the link https://osf.io/egqaz. Or on the OSF wiki page, it is under the tab 'registrations'. The project was registered on 26th July 2021. The authors have also attached a pdf version of the protocol registration for ease of access.

2c. As the authors argue, too low complexity of music can lead to boredom, and too high complexity of the music can lead to irritation or over-stimulation (lines 92-94). It can be assumed that the optimal level of complexity would be between low and high complexity. Still, the authors decided to only investigate low and high complexity which might both not lead to optimal results. It is still interesting to investigate the two rather extreme forms of complexity, but the missing “moderate” complexity condition might be a limitation of the study or eventually one possible explanation for not having found an effect for complexity, and could be discussed later in the manuscript.

**Response:** Optimal complexity is a relative term. We used the terms “high” and “low” to describe the conditions relative to each other. The section on “Musical Stimuli” details our thinking behind the design of these conditions. Working with the music composer, the “high complexity” track was designed to lead to sustained engagement and enjoyment from a general audience across all age groups. While it was high relative to the low condition, we can distinguish it from one that is “too high” to be enjoyable to a general audience. We could rename the conditions as “moderate complexity” vs “simple” but we have retained our naming in the revised manuscript for the sake of simplicity. Nevertheless, tis has been clarified in Lines 279 and 280.

3. The methods section is now more elaborated, and important paragraphs have been included. One issue still remains to be considered. The percent values of the distribution of the participants (lines 176-177) across Europe (37.41%) and the United States (36%) don’t sum up to 100%, so the question arises if also other continents are involved or if the percent values should be adapted. Also, the other percent values regarding the different types of pain seem to be not completely correct. These percent values should be re-checked.

**Response:** Additional details of the geographical distribution of participants have been included 194-195. The values representing pain are frequency values rather than percentages so they sum to the total number in the sample. All of the values have been re-checked and one value was found to need correction so this was updated.

4. The results are now clearer and very informative details on multilevel analyses are given. Some remaining issues concern mainly the discussion part. In general, some parts of the hypotheses should be answered in more detail in the discussion part. It could be considered to discuss at first the results regarding H1, then H2, then H3, and then findings on the profile of individual characteristics. The following issues should be considered:

4a. The authors write that H1 would be supported by having replicated the analgesic effects of cognitive agency which demonstrated the benefits of choosing music in a pain context (lines 387-389). H1 targets both pain intensity and pain unpleasantness, but choice only had an effect on pain intensity in the depicted results. This should also become clear in the discussion part by mentioning and discussing that the hypothesized effect was confirmed for pain intensity but not pain unpleasantness.

**Response** Line 436-437 have been amended to report the findings with more detail.

4b. Similarly, the authors discuss the sig. interaction effect between choice and active engagement (lines 421-427). It should also be mentioned and discussed that this interaction effect was found to be sig. for pain intensity but not pain unpleasantness.

**Response** Line 478-480 have been amended to report the findings with more detail.

4c. The authors conclude that the findings suggested that the cognitive mechanisms outlined by the cognitive vitality model could meaningfully impact acute pain in day-to-day living (lines 437-439). Keeping in mind that the hypotheses were only partly confirmed (see also 4a and b), this conclusion should be formulated in a more differentiated or cautious way. For the same reason, some of the following conclusions and discussions on the analgesic effects of active engagement and choice should rather target specific aspects of pain than pain in general, also in the part “Summary and Conclusions”.

**Response** Line 491-492 and the summary and conclusion have been re-formulated in a more differentiated way.

4d. The authors write that in a real-world setting, it would not be possible to recreate the feeling of acute pain once it has been decreased by music engagement (lines 449-450). Since acute pain can sometimes re-occur after some time, I recommend to change the formulation of the sentence.

**Response:** Line 503-505 have been reformulated to more precisely express the point.

4e. Keeping in mind that the hypotheses are only partly confirmed regarding the effect of choice on different pain outcomes, that the eligibility criteria were not so many, that different types of pain were mixed in the study, that a between-subjects design was used and the hypotheses have not yet been investigated in a therapeutic context, it is too early to give the recommendation of letting clients select music themselves in a therapeutic context (lines 461-463).

**Response:** Lines 514-517 have been revised.

4f. I highly appreciate that the effort was made to compose new music for the study. It should be considered to discuss in the discussion that the complexity conditions differed in tempo also.

**Response** The impact of the additional ornamentation and percussion on the perceived tempo has been highlighted from line 468-472.

5. The new title lets assume that agency and active engagement predicted pain decreases in the different pain outcomes, which has been found only partly. The title would be improved by formulating it in a way that represents more results, for example by writing “the role of…” instead of “predict”.

**Response:** The title has been amended to reflect that the outcomes have been found partly.

6. The authors made clearer in the introduction and the discussion that the results and previous findings can only be interpreted for investigated music features. In the abstract it still says “Overall, findings demonstrated that the illusion of choice has analgesic benefits, and that perceived choice is more important than music features” (lines 28-29). The authors should make clear that this can be concluded only for the “investigated” or “selected” or “specific” music features, or write about “complexity/ (eventually tempo)” instead of “music features” in general.

**Response** The abstract has been amended to specifically refer to music complexity.

7. In line 452 it should say that there is “no” group with no music as a control.

**Response:** Line 505 has been amended.

8. The manuscript should be re-checked regarding typos and small grammar mistakes (for example l. 103: „emotions“ instead of „emotion’s”, l. 133: “ensure” better than “insure”, l. 238: “Barcelona Musical Reward” can probably be deleted, or should be specified otherwise, l. 348: “.” is missing, l. 356 & 357: “were”).

**Response:** The document has been carefully revised and corrected for typos.

Reviewer #2: Thank you to the authors for revising the manuscript. While much of the issues were clarified and resolved, I am still very concerned about the logic and the reporting of the statistical analysis. Specifically:

1. I still do not understand the merit of using mixed models in an independent samples design. The authors state that model fit was not improved by including a random factor for participants, "Therefore, random factors were excluded from the final models.". If by random factors authors mean random effects (in R terminology), why even consider them if there are no repeated measures for participants? A regular linear model seems an obvious choice in this experimental design. Using it would also enable the authors to quantify the variance explained by their models using (easily-interpretable) R^2 statistics.

**Response** The core reason for using a multilevel model in an independent samples design is to account for the hierarchical structure in the data, which can be empirically identified by the presence of an intraclass correlation coefficient of over 0.2. In the current study an ICC of 0.67 is identified which demonstrates that the residuals are not independent, which precludes the use of a regression analysis based on an ordinary least squares calculation. An additional reason for using a multilevel model in this context is because it allows you to simultaneously examine higher order effects from lower order effects, which means you can disentangle which effect is accounting for more of the variance. The paragraph that highlights the rationale for using multi-level modelling has been further amended to highlight this, and includes further citations to support this approach line 274 - 286.

2. What do the numbers presented in Tables 3 and 4 stand for? Are they model estimates for each of the predictors? If so, they should be labelled as such.
**Response** Additional labels have been added in table 3 and 4.

3. I do not understand the rationale behind "A top-down linear modelling strategy with a loaded mean structure" (L284). How exactly was the initial model specified? Did the results of the initial model reveal the same significant effects as the final model? If so, why remove the non-significant effects? If not, can the authors explain why? How does this influence the interpretation of the results? Why age was included and not other demographic variables, such as gender? Were all possible interactions explored? If so, why? If not, why? Was the procedure automated? Assuming this approach is similar to step-wise regression procedures, when it was set to stop (that is, which model was considered the most "parsimonious")? Was this decision based on log likelihood or on "removing non-significant effects" (L286)? What was the criterion for "non-significant effect"?

**Response:** The reporting of the results has been re-written and re-organised to facilitate a clearer representation of the final results. The rationale for using the linear model is detailed in the data analysis section in the method, and the three step process used for the linear modelling approach has been outlined for each of the two dependant variables, and this is reported in the results section. Additional citations have been provided that outline the rationale for using multilevel modelling with independent samples.

4. Also, I am not sure if this approach is necessary, or even valid here. There are clear hypotheses and a simple experimental design. Including all measured individual difference variables in the model could be beneficial, as it would account for any variance arising from these variables (even if they are "not significant"). This would then enable more robust judgments about the presence (or lack thereof) of main effects and interactions. The authors used what looks like an exploratory approach (although it is hard to tell for certain) that is rarely seen in experimental studies and does not seem to be justified.

**Response:** The core reason for using a multilevel model in an independent samples design is to account for the hierarchical structure in the data, which can be empirically identified by the presence of an intraclass correlation coefficient of over 0.2. In the current study an ICC of 0.67 is identified which demonstrates that the residuals are not independent, which precludes the use of a regression analysis based on an ordinary least squares calculation. An additional reason for using a multilevel model in this context is because it allows you to simultaneously examine higher order effects from lower order effects, which means you can disentangle which effect is accounting for more of the variance. The paragraph that highlights the rationale for using multi-level modelling in the data analysis section of the method has been further amended to highlight this, and the authors have included further citations to support this approach line 274 - 286.

5. Finally, the way results are presented is puzzling and unclear. For example, the authors state "significant main effects of both choice, F(1, 269) = 4.82; p < .05, and active engagement, F(1, 272) = 4.21; p < .05.". Why F-test results are given? What are these test actually veryfing? If they were testing the significance of main effects (or, perhaps more accurately, model parameters as this is linear modelling), why not use the t-test? Why do degrees of freedom seem to vary between different parameters of the same model? And crucially, where are the parameter estimates reported?

**Response:** The reporting of the results has been re-written and re-organised to facilitate a clearer representation of the final results. The rationale for using the linear model is detailed in the data analysis section in the method, and the three step process used for the linear modelling approach has been outlined for each of the two dependant variables. F statistics have been removed for the models, and confidence intervals have been included with the parameter estimates. Additional citations have been provided that outline the rationale for using multilevel modelling with independent samples.

All these issues may potentially bias the results, therefore it is hard to judge the conclusions of this study without claryfing the statistical issues first.

Other issues:

- The added sections and clarifications contain some grammatical errors that need to be correted. I would suggest a thorough proof-reading of the manuscript.

**Response** The document has been carefully revised and corrected for typos.

- Figure 1 appears to be missing.

**Response** Figure 1 is submitted as a separate document and appears at the end of the document.

- Describing the track comparisons in terms of "Hypothesis 3" is a bit misleading, as these comparisons are in fact manipulation checks and not verifications of a hypothesis derived from theory. I understand that this is related to pre-registration, but I would suggest not describing these as "H3" in the text.

**Response** The final analysis is no longer described as a 'hypothesis 3' to aid understanding.

- L166-167: "In stage one 2691 answered screening questions which were presented one at a time, based on each previous response along with red herring questions to reduce the likelihood that participants would guess the nature of the study." It is unclear what "2691" refers to.

**Response** Line 183 has been amended.

- The authors indicate that the data from the study will be made publicly available in the OSF, yet the repository is empty as of writing this review.

**Response**: There seems to be a technical issue with the OSF repository, on our side we can see the review at the link https://osf.io/egqaz . Or on the OSF wiki page, it is under the tab 'registrations'. The project was registered on 26th July 2021. The authors have also attached a pdf version of the protocol registration for ease of access.
