# Supplementary material for: Tune out pain: Agency and active engagement predict decreases in pain intensity after music listening
Source: PLoS One. 2022 Aug 3;17(8):e0271329. doi: 10.1371/journal.pone.0271329 (PMC9348657; doi:10.1371/journal.pone.0271329)
Supplement: Attachment — Submitted filename: Response to reviewers.docx [file pone.0271329.s004.docx]

Response to reviewers.

We thank each of the reviewers, and the editor, for their time and attention on this manuscript. In response to the final comments from reviewers, we have added further clarification in relation to the statistical analysis and discussion.

Additionally, one of the reviewers is quite right to point out that there is a difference between mixed models, and hierarchical models. They can overlap, but there is a difference, and it is important to get that right. We hope that you will agree that the manuscript has been further strengthened it terms of clarity, and should be of high interest to the readers of PLoS one.

Below you will find a point by point response to each of the queries raised.

1. "A threshold of p < 0.20 for the LR χ2 (2) was used for each model comparison" Why p < .20? There should be some justification to this. Would a smaller p value lead to different parameter selection? This is important is it may potentially influence the results.

**Response:** The following text was added to provide a justification for this threshold:

"A threshold of *p* < 0.20 for the LR χ² (2), was chosen based on previous recommendations (47,55) to balance between the risk of a type 1 error that may occur with an overly inclusive approach to modelling (e.g. a maximalist approach), and a parsimonious approach to modelling that would tend to *exclude* more parameters."

2. What does B stand for in the Results? (L424-425) If it is the model estimate, it would probably be more appropriate to use Beta (ß) instead.

**Response:** ß has been inserted to report the model estimate.

3. L437: "Supporting Hypothesis 1, ...". It seems that this hypothesis was only partly supported, given that there was no significant effect on pain unpleasantness. (L132-133: "Here we predict that increased perceived control predicts decreases in pain intensity and pain unpleasantness (H1)."). The authors should address this in the discussion.

**Response:** The revised manuscript includes the following text on line 483- 485:

"However, it is important to note that the main effect of cognitive agency, and the interaction between cognitive agency and active engagement were only observed in relation to decreases in pain intensity and not pain unpleasantness."
